# Maternal mental health and gestational weight gain in a Brazilian Cohort

**DOI:** 10.1038/s41598-021-90179-6

**Published:** 2021-05-24

**Authors:** Dayana Rodrigues Farias, Thais Rangel Bousquet Carrilho, Nathalia C. Freitas-Costa, Mônica Araújo Batalha, Mylena Gonzalez, Gilberto Kac

**Affiliations:** Nutritional Epidemiology Observatory, Department of Social and Applied Nutrition, Josué de Castro Institute of Nutrition, Federal University of Rio de Janeiro, Avenida Carlos Chagas Filho, 373, CCS, Bloco J, 2º andar, sala 29. Cidade Universitária – Ilha do Fundão, Rio de Janeiro, RJ 21941-902 Brazil

**Keywords:** Nutrition, Weight management, Epidemiology

## Abstract

Depression and anxiety are common during pregnancy, but little is known about the influence of these disorders on gestational weight gain (GWG). Data from a prospective cohort of pregnant women followed in a public healthcare center in Rio de Janeiro, Brazil, were used to evaluate the association of depression, anxiety, and suicide risk with GWG. GWG was evaluated at 5–13, 20–26, 30–36, and 37–42 weeks, and GWG adequacy was determined. Statistical analyses included linear mixed-effect models and Poisson regression. We evaluated 206 women, in which 15% (n = 31) presented major depressive disorder, 19.4% (n = 34) suicide risk and 10% (n = 21) generalized anxiety disorder at baseline. Women with depression at the first trimester, persistent depressive symptoms, and anxiety symptoms at the second trimester presented significantly lower rates of GWG per week compared to those without depression or anxiety, respectively. Persistent depressive symptoms represented a 2.40 (95% CI 1.20; 4.81; p = 0.013) increase in the risk of insufficient GWG. There was no significant association between generalized anxiety disorder or suicide risk with GWG. The presence of depression, depressive symptoms, and anxiety during pregnancy were associated with lower GWG rates. Persistent depressive symptoms during pregnancy were directly associated with insufficient GWG.

## Introduction

Mental health disorders such as depression and anxiety are common during pregnancy^1,2^. The prevalence of depression and anxiety vary widely depending on the pregnancy period and country and has been reported to be between 7 and 22% worldwide^3–7^. In low- and middle-income countries, this prevalence can be higher^8,9^. Studies during pregnancy conducted in Brazil revealed prevalence around 14% to 20% for depression^10–12^ and 19% to 40% for anxiety^13–15^. The prevalence of suicide intention or suicide ideation varies from 5 to 14%^16,17^ worldwide, and Brazilian studies reported values between 18^18^ and 23%^19^ during pregnancy.

These mental health disorders are a significant public health concern and may have short- and long-term impacts on the mother’s and offspring’s life^20–23^. A systematic review revealed that depressive symptoms during pregnancy were associated with preterm birth and low birth weight^24^. Maternal mental health may modify appetite^25^, dietary quality^26,27^, and physical activity^28,29^, which can also contribute to weight changes, and more specifically, lead to inadequate gestational weight gain (GWG)^6,30–32^.

GWG is an important and potentially modifiable risk factor for several maternal and child health outcomes^33,34^. Maternal weight gain trajectory was associated with increased risk of small-for-gestational-age (SGA) birth^35^ and offspring obesity^36^. GWG above or below the American Institute of Medicine (IOM) guidelines^34^ is also related to gestational diabetes, hypertensive disorders, postpartum weight retention, and SGA birth and large for gestational age (LGA) and preterm infants^34,37,38^.

Maternal mental health status during pregnancy may play a role in the determination of GWG. Two systematic reviews have evaluated the relationship between maternal psychological distress, including depression and anxiety, and GWG. However, the results are still inconclusive^31,39^. In addition, the effect of these disorders on GWG trajectories remains unclear, especially in low and middle-income countries, where the prevalence of those disorders has increased substantially^8,9^. Thus, this study aimed to estimate the association between depression, anxiety, and suicide risk with GWG trajectories and GWG adequacy among Brazilian women.

## Results

The study had 8.2% of losses to follow-up, and we did not find significant differences in sociodemographic, obstetric, and anthropometric characteristic or mental health status at the study baseline between women who completed the follow-up (n = 189) and those who were lost (n = 17) (Supplemental Table 1).

Fifteen percent (n = 31) of the women had major depressive disorder, 19.4% (n = 34) suicide risk, and 10% (n = 21) generalized anxiety disorder at the study baseline. The prevalence of depressive symptoms decreased from 34.6% in the first trimester to 20.9% in the third trimester, and 9.1% (n = 16) presented persistent depressive symptoms during pregnancy. Most women presented GWG above the IOM guidelines at the third trimester (53.7%, n = 94), and 19.7% (n = 38) presented GWG below it (Table 1).

**Table 1:** Mental health status, socioeconomic and demographic characteristics according to GWG adequacy at the third trimester.

Maternal characteristics	N (%)	GWG adequacy^a^			*p*^c^	GWG^b^ (kg)	
		Adequate	Insufficient	Excessive		Mean (SD)	*p*^d^
		n (%)	n (%)	n (%)			
	188	52 (27.7)	37 (19.7)	99 (52.6)		13.2 (5.5)	
**First-trimester major depressive disorder**^**e**^							
No	163 (86.7)	44 (84.6)	30 (81.1)	89 (89.9)		13.5 (5.6)	
Yes	25 (13.3)	8 (15.4)	7 (18.9)	10 (10.1)	0.352	10.9 (4.9)	0.028
**Generalized anxiety disorder**^**e**^							
No	169 (89.9)	43 (82.7)	34 (91.9)	92 (92.9)		13.3 (5.7)	
Yes	19 (10.1)	9 (17.3)	3 (8.1)	7 (7.1)	0.126	12.1 (4.4)	0.400
**Suicide risk**^**e**^							
No	152 (80.6)	43 (82.7)	28 (75.7)	81 (81.8)		13.3 (5.5)	
Yes	34 (19.4)	9 (17.3)	9 (24.3)	18 (18.2)	0.666	12.5 (5.7)	0.420
**First-trimester EPDS score**							
< 11	122 (65.6)	29 (55.8)	23 (63.9)	70 (71.4)		13.7 (5.3)	
≥ 11	64 (34.4)	23 (44.2)	13 (36.1)	28 (28.6)	0.153	12.2 (5.7)	0.067
**Second-trimester EPDS score**							
< 11	138 (78.9)	36 (73.5)	23 (69.7)	79 (85.0)		13.8 (5.4)	
≥ 11	37 (21.1)	13 (26.5)	10 (30.3)	14 (15.0)	0.101	11.1 (5.7)	0.010
**Third-trimester EPDS score**							
< 11	145 (78.4)	36 (69.2)	28 (75.7)	81 (84.4)		13.6 (5.6)	
≥ 11	40 (21.6)	16 (30.8)	9 (24.3)	15 (15.6)	0.092	11.2 (5.1)	0.014
**Persistent depressive symptoms**^**f**^							
No	153 (90.5)	41 (83.7)	26 (81.3)	86 (97.7)		13.6 (5.4)	
Yes	16 (9.5)	8 (16.3)	6 (18.7)	2 (2.3)	0.002	8.6 (4.9)	< 0.001
**Second trimester state anxiety score**^**g**^							
< 40	104 (58.4)	28 (57.1)	14 (41.2)	62 (65.3)		14.0 (5.6)	
≥ 40	74 (41.6)	21 (42.9)	20 (58.8)	33 (34.7)	0.049	12.0 (5.4)	0.015
**Third trimester state anxiety score**^**g**^							
< 40	98 (53.0)	20 (39.2)	21 (56.8)	57 (58.8)		13.4 (5.8)	
≥ 40	87 (47.0)	31 (60.8)	16 (43.2)	40 (41.2)	0.067	12.9 (5.5)	0.538
**Persistent anxiety symptoms**^**f**^							
No	122 (70.1)	29 (60.4)	21 (61.8)	72 (78.3)		13.7 (5.9)	
Yes	52 (29.9)	19 (39.6)	13 (38.2)	20 (21.7)	0.045	11.9 (4.8)	0.050
**Age (years)**							
< 30	127 (67.6)	37 (71.2)	26 (70.3)	64 (64.6)		12.9 (5.6)	
≥ 30	61 (32.4)	15 (28.8)	11 (29.7)	35 (35.4)	0.666	13.6 (5.4)	0.383
**Education (schooling years)**							
< 8	58 (30.8)	16 (30.8)	14 (37.8)	28 (28.3)		12.8 (5.4)	
≥ 8	130 (69.2)	36 (69.2)	23 (62.2)	71 (71.7)	0.562	13.3 (5.6)	0.630
**Marital status**							
Live with partner	152 (80.8)	42 (80.8)	31 (83.8)	79 (79.8)		13.2 (5.5)	
Does not live with partner	36 (19.2)	10 (19.2)	6 (16.2)	20 (20.2)	0.871	13.8 (5.9)	0.682
**Parity (parturitions)**							
0 or 1	143 (76.1)	39 (75.0)	31 (83.8)	73 (73.7)		12.7 (5.6)	
≥ 2	45 (23.9)	13 (25.0)	6 (16.2)	26 (26.3)	0.165	14.5 (5.2)	0.055
**Desire to be pregnant**							
Yes	76 (40.4)	24 (46.2)	16 (43.2)	36 (36.4)		12.4 (5.7)	
No	112 (59.6)	28 (53.8)	21 (56.8)	63 (63.6)	0.470	13.6 (5.4)	0.155
**Pre-pregnancy BMI (kg/m**^**2**^**)**							
< 25	117 (62.2)	37 (71.2)	25 (67.6)	55 (55.6)		13.8 (5.0) a	
25–29.9	50 (26.6)	12 (23.1)	8 (21.6)	30 (30.3)		12.4 (6.0) a,b	
≥ 30	21 (11.2)	3 (5.8)	4 (10.8)	14 (14.1)	0.310	10.7 (6.4) b	0.024
**Pre-pregnancy leisure time physical activity**							
No	140 (74.5)	38 (73.1)	32 (86.5)	70 (70.7)		12.7 (5.6)	
Yes	48 (25.5)	14 (26.9)	5 (13.5)	29 (29.3)	0.165	14.5 (5.2)	0.055

*GWG* gestational weight gain, *EPDS* Edinburgh Postnatal Depression Scale, *BMI* body mass index, *SD* standard deviation, *N* sample size.

^a^GWG adequacy according to the Institute of Medicine (2009) guidelines for gestational week; ^b^GWG measured at the third trimester [median 39 (IQR: 38; 40) gestational weeks]; ^c^p-value refers to chi-squared test; ^d^p-value refers to Student’s *t* test or one-way ANOVA. ^e^Mental health status assessed using Mini-International Neuropsychiatric Interview. ^f^Persistent depressive symptoms were classified as ‘yes’ if the women presented Edinburgh Postnatal Depression Scale score ≥ 11 at the three pregnancy trimesters. ^g^State anxiety symptoms were measured using the Spielberger State-Trait Anxiety Inventory. ^f^Persistent anxiety symptoms were classified as ‘yes’ if the women presented Spielberger State Anxiety Inventory score ≥ 40 at the second and third trimesters. The analyses were not adjusted.

Women with first-trimester depression presented a significantly lower mean of total GWG compared to women without depression (10.9 (SD = 4.9) vs. 13.5 (SD = 5.6); p = 0.028]. The presence of depressive symptoms at second (11.1 (SD = 5.7) vs. 13.8 (SD = 5.4); p = 0.010] and third [11.2 (SD = 5.1) vs. 13.6 (SD = 5.6); p = 0.014] trimester and persistent depressive symptoms during pregnancy [8.6 (SD = 4.9) vs. 13.6 (5.4); p < 0.001] were associated with lower means of total GWG. It was observed a higher proportion of insufficient GWG in women with persistent depressive symptoms than adequate or excessive gestational weight gain [18.7% vs. 16.3% and 2.3%; p = 0.002, respectively]. Women classified as having anxiety symptoms at the second trimester presented significant lower means of total GWG [12.0 (SD = 5.4) vs. 14.0 (SD = 5.6); p = 0.015] and a higher proportion of insufficient GWG, compared to those with adequate or excessive GWG (58.8% vs. 42.9% and 34.7%; p = 0.049, respectively) (Table 1).

**Figure 1:**
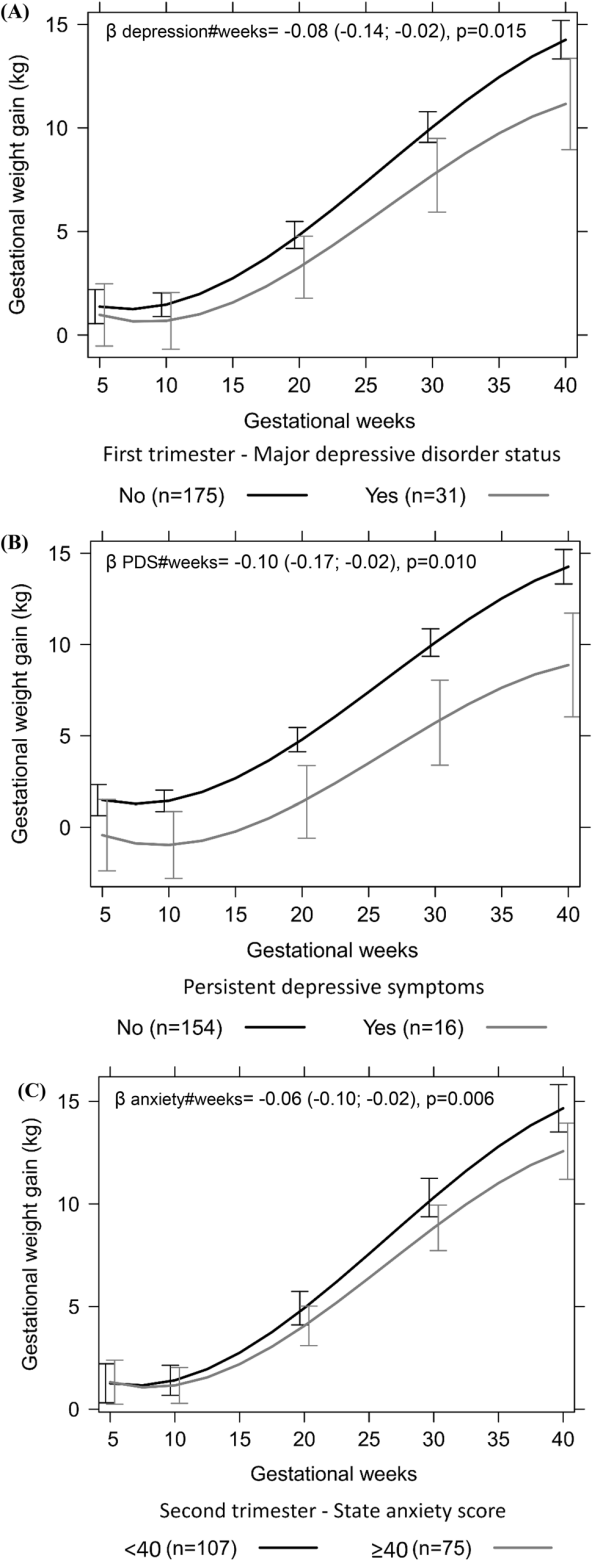
Longitudinal prediction of gestational weight gain trajectory according to (**A**) first-trimester major depressive disorder status, (**B**) persistent depressive symptoms (PDS), and (**C**) Second-trimester state anxiety score. All the models were constructed using cumulative gestational weight gain and adjusted for linear, quadratic, and cubic gestational weeks, desire to be pregnant, education, marital status, pre-pregnancy leisure time physical activity, parity, and pre-pregnancy body mass index. (**A**) Number of observations: 749; Number of women: 206; Average of 3.6 observations per woman. Major depressive disorder status was assessed using Mini-International Neuropsychiatric Interview. (**B**) Number of observations: 645; Number of women: 165; Average of 3.9 observations per woman. Persistent depressive symptoms were classified as ‘yes’ if the women presented Edinburgh Postnatal Depression Scale score ≥ 11 at the three pregnancy trimesters. (**C**) Number of observations: 707; Number of women: 182; Average of 3.9 observations per woman. Anxiety symptoms were measured using the Spielberger State Anxiety Inventory.

We observed a significant difference in GWG trajectory in women with depression in the first trimester compared with those without depression diagnostic, in which depressive women presented a lower rate of GWG per gestational week and a significant lower mean of cumulative GWG in the third trimester (Table 2 and Fig. 1A). Women with persistent depressive symptoms during pregnancy presented a lower rate of GWG per gestational week and a significantly lower mean of cumulative GWG in the second and third trimester, compared to those without persistent depressive symptoms (Table 2 and Fig. 1B). We observed a similar association between second-trimester state anxiety and GWG. Still, we did not observe significant differences in mean cumulative GWG between women with and without second-trimester state anxiety symptoms (Table 2 and Fig. 1C). Those associations were not attenuated by the adjustment for the minimal set of confounders (Table 2 and Fig. 1A–C). It was also observed that persistent depressive symptoms represented a 2.40 (95% CI 1.20; 4.81; p = 0.013) increase in the risk of insufficient GWG (Table 3).

**Table 2:** Association between maternal mental health during pregnancy and gestational weight gain for women followed at a public health care center in Rio de Janeiro, Brazil, 2009–2012.

	Gestational weight gain^a^ (kg, n = 206)			
	Model 1		Model 2	
	*β *(95% CI)^b^	*p-*value^c^	*β *(95% CI)^b^	*p-*value^c^
**First trimester major depressive disorder (no/yes)**	0.15 (− 1.43; 1.73)	0.854	− 0.04 (− 1.63; 1.55)	0.959
Gestational weeks	− 0.48 (− 0.77; − 0.19)	0.001	− 0.46 (− 0.76; − 0.18)	0.002
Quadratic gestational weeks	0.04 (0.02; 0.05)	< 0.001	0.04 (0.02; 0.05)	< 0.001
Cubic gestational weeks	− 0.0005 (− 0.0007; − 0.0003)	< 0.001	− 0.0005 (− 0.0007; − 0.0003)	< 0.001
Interaction term^d^				
Major depressive disorder#gestational weeks	− 0.08 (− 0.14; − 0.02)	0.008	− 0.08 (− 0.14; − 0.02)	0.015
**Persistent depressive symptoms (no/yes)**	− 1.40 (− 3.42; 0.62)	0.173	− 1.27 (− 3.31; 0.76)	0.220
Gestational weeks	− 0.54 (− 0.84; − 0.24)	< 0.001	− 0.54 (− 0.84; − 0.24)	< 0.001
Quadratic gestational weeks	0.04 (0.03; 0.06)	< 0.001	0.04 (0.03; 0.06)	< 0.001
Cubic gestational weeks	− 0.0005 (− 0.0007; − 0.0003)	< 0.001	− 0.0005 (− 0.0007; − 0.0003)	< 0.001
Interaction term^d^				
Persistent depressive symptoms#gestational weeks	− 0.10 (− 0.17; − 0.02)	0.011	− 0.10 (− 0.17; − 0.02)	0.010
**Second-trimester anxiety symptoms (no/yes)**	0.44 (− 0.76; 1.65)	0.474	0.37 (− 0.84; 1.58)	0.381
Gestational weeks	− 0.47 (− 0.77; − 0.18)	0.002	− 0.46 (− 0.76; − 0.16)	0.002
Quadratic gestational weeks	0.04 (0.02; 0.05)	< 0.001	0.04 (0.02; 0.05)	< 0.001
Cubic gestational weeks	− 0.0005 (− 0.0007; − 0.0003)	< 0.001	− 0.0005 (− 0.0007; − 0.0003)	< 0.001
Interaction term^d^				
State anxiety score #gestational weeks	− 0.07 (− 0.11; − 0.02)	0.005	− 0.06 (− 0.10; − 0.02)	0.006

Major depressive disorder was assessed using Mini-International Neuropsychiatric Interview. Persistent depressive symptoms were classified as ‘yes’ if the women presented Edinburgh Postnatal Depression Scale score ≥ 11 at the three pregnancy trimesters. State anxiety symptoms were measured using the Spielberger State-Trait Anxiety Inventory. Model 1 was adjusted for linear, quadratic, and cubic gestational weeks; Model 2 was further adjusted for desire to be pregnant, education, marital status, pre-pregnancy leisure time physical activity, parity, and pre-pregnancy body mass index.

^a^Cumulative gestational weight gain; ^b^Longitudinal linear regression coefficient and 95% confidence interval (CI); ^c^p-value refers to maximum likelihood estimator; ^d^The interaction term between mental health status and gestational weeks tests whether there were different trajectories of gestational weight gain according to the categories of the mental health status variable.

**Table 3:** Association between maternal mental health during pregnancy and insufficient gestational weight at the third trimester for women followed at a public health care center in Rio de Janeiro, Brazil, 2009–2012.

	Insufficient gestational weight gain^a^			
	Model 1		Model 2	
	IRR (95% CI)	*p-*value^b^	IRR (95% CI)	*p-*value^b^
First trimester major depressive disorder (no/yes)	1.52 (0.75; 3.09)	0.246	1.64 (0.80; 3.37)	0.172
Persistent depressive symptoms (no/yes)	2.21 (1.07; 4.56)	0.032	2.40 (1.20; 4.81)	0.013
Second trimester anxiety symptoms (no/yes)	1.70 (0.93; 3.10)	0.081	1.77 (0.97; 3.24)	0.064
Third trimester anxiety symptoms (no/yes)	1.02 (0.55; 1.87)	0.939	1.01 (0.55; 1.86)	0.972
Generalized anxiety disorder (no/yes)	0.78 (0.26; 2.32)	0.661	0.85 (0.29; 2.47)	0.762
Suicide risk (no/yes)	1.36 (0.70; 2.62)	0.364	1.58 (0.81; 3.08)	0.175

Major depressive disorder, generalized anxiety disorder, and suicide risk were assessed using Mini-International Neuropsychiatric Interview. Persistently depressive symptoms were classified as yes if the women presented Edinburgh Postnatal Depression Scale score ≥ 11 at the three pregnancy trimesters. State anxiety symptoms were measured using the Spielberger State-Trait Anxiety Inventory. Model 1 was not adjusted; Model 2 was adjusted for desire to be pregnant, education, marital status, pre-pregnancy leisure time physical activity, parity, and pre-pregnancy body mass index.

*CI* confidence interval, *IRR* Incidence rate ratio.

^a^Gestational weight gain adequacy according to Institute of Medicine guidelines (adequate/excessive vs. insufficient weight gain); ^b^p-value refers to Poisson regression.

We did not observe a statistically significant association between generalized anxiety disorder or suicide risk with mean, trajectory (data not shown in tables), or adequacy of GWG in crude or adjusted models (Table 3).

## Discussion

The study main finding was that women with persistent depressive symptoms during pregnancy presented a 0.1 kg/week lower rate of GWG throughout pregnancy and a 140% higher risk of insufficient GWG than women without persistent symptoms. The presence of major depressive disorder in the first trimester and second-trimester anxiety symptoms was also associated with a 0.08 kg/week and a 0.06 kg/week lower rate of GWG, respectively.

To the best of our knowledge, this is the first study that evaluated the association of maternal mental health prospectively measured in different time points during pregnancy, i.e., at the three pregnancy trimesters and GWG trajectories. The main strengths of this study are the longitudinal design that made it possible to evaluate GWG trajectories and repeated measures of maternal mental health during pregnancy. We also stand out using different methods (a diagnostic interview and screening scales) to evaluate maternal mental health. Furthermore, the use of LME models allowed women to include at least one measure of exposure and outcome during pregnancy. Thus women with missing data were not completely excluded from the analysis.

In contrast, the current study has limitations that are worth mentioning. First, like most longitudinal studies, the losses of follow-up (8.2%) reduced our sample size across time. However, we did not find significant differences between those who completed the study and the follow-up losses. We used self-reported pre-pregnancy weight to calculate GWG, which could introduce bias to our analyses. Nevertheless, studies have shown that women report pre-pregnancy weight accurately. The pre-pregnancy BMI classification with self-reported or first-trimester measured weight presents high agreement, especially in Brazil^40–43^.

We found that persistent depressive symptoms were associated with lower GWG during pregnancy and increased risk of insufficient total GWG. The diagnosis of depression in the first trimester was also associated with lower GWG. Badon et al.^6^ evaluated 79,506 women from Northern California (USA), and found a U-shape association, in which maternal depression at any time 6 months prior to pregnancy was associated with 11% greater risk of GWG rate below the IOM guideline and 3% greater risk of GWG rate above the IOM guideline. The early pregnancy onset depression (up to 20 gestational weeks) was associated with a 4% higher risk of excessive GWG than non-depressed women. However, different studies did not find significant associations or found it in a different direction. Molyneaux et al.^3^ evaluated 13,314 pregnant women from the Avon Longitudinal Study of Parents and Children (UK) and did not find significant associations between persistent depressive symptoms (measured using EPDS at 18 and 32 weeks) with either inadequate or excessive GWG. Dolin, et al.^44^ evaluated 508 low-income Hispanic women and did not find significant associations between second-trimester depressive symptoms and total GWG. Braig, et al.^45^ used data from a birth cohort study in Ulm, Germany, to associate maternal depressive symptoms and GWG in 718 women. The authors did not find significant associations between depressive symptoms and GWG adequacy or GWG trajectories. Matthews et al.^32^ conducted a cross-sectional study with 1073 USA pregnant women and found a positive association between depressive symptoms and GWG at the second trimester. Hence, it is possible to observe no consensus about the association between maternal depression or depressive symptoms and GWG. The studies used different methodological procedures, such as scales to evaluate depressive symptoms and measurements taken at other time points during pregnancy and different study designs, making comparisons difficult. The study of Molyneaux, et al.^3^ had an elevated sample size, but the authors evaluated persistent depressive symptoms in a short time compared to our study. Braig, et al.^45^ measured depressive symptoms retrospectively reported after birth, which could have introduced bias due to the women’s memory and/or her current mental health status. Finally, although Matthews et al.^32^ evaluated trimester-specific associations during pregnancy, the cross-sectional design did not allow the authors to model the GWG trajectories.

We also found that women with anxiety symptoms measured at the second trimester presented lower rates of GWG during pregnancy compared to those without anxiety symptoms. However, anxiety symptoms were not associated with GWG below IOM guidelines. Vehmeijer, et al.^4^ used data from a population-based cohort of 3,393 pregnant women and found that those with anxiety symptoms had a 40% lower risk of excessive weight gain, but the symptoms were not associated with increased risk of insufficient GWG, a result in line with the current study. On the other hand, Braig, et al.^45^, did not find significant associations between anxiety symptoms and GWG trajectories during pregnancy in a sample of German women. The distinction between the results of Braig, et al.^45^ and ours may be attributed to differences in the scale used to assess anxiety symptoms and the fact that in their study, anxiety was retrospectively reported after delivery. Although Vehmeijer et al.^4^ used a different scale to estimate anxiety symptoms, they also evaluated it at the second trimester of pregnancy.

The mechanisms underlying the association of depression and anxiety with inadequate GWG are complex, but there is increasing evidence on depression and anxiety on dietary intake. Women with depressive symptoms tend to present a lower intake of macronutrients (carbohydrates, proteins, and fats) and poor diet quality than those without symptoms^46,47^. Anxiety has also been reported to affect dietary intake during pregnancy by increasing fat intake and decreasing micronutrient intake^48^. A study conducted by Din, et al.^49^ found that women with depression, anxiety, and stress consumed less variety of foods and had lower intakes of milk, meat, and fruit. Thus, we hypothesized that the association of depression and anxiety with insufficient GWG is mediated by inadequate dietary intake during pregnancy. The evaluation of this possible mediation path in future studies may explain the observed association between depression, anxiety, and GWG in this study.

In conclusion, we found that maternal depression and anxiety symptoms were associated with insufficient GWG. The evaluation of mental health is not part of the prenatal care routine in the Brazilian Unified Health System. Still, the results of the present study suggest that the evaluation of depressive and anxiety symptoms during pregnancy should be considered to screen women at risk of insufficient GWG. This is the first study evaluating the longitudinal association between maternal depressive and anxiety symptoms and GWG. Although our results point towards a clinically meaningful association, we highlight that one should keep in mind that these results still need to be confirmed by studies with larger sample sizes and different populations.

## Methods

### Study design and sample

We used data from a prospective cohort of apparently healthy pregnant women evaluated during routine prenatal care in a public healthcare center in Rio de Janeiro, Brazil. The enrollment occurred from November 2009 to October 2011, and the present study analyzed data of four gestational time points (5–13, 20–26, 30–36, and 37–42 weeks).

**Figure 2:**
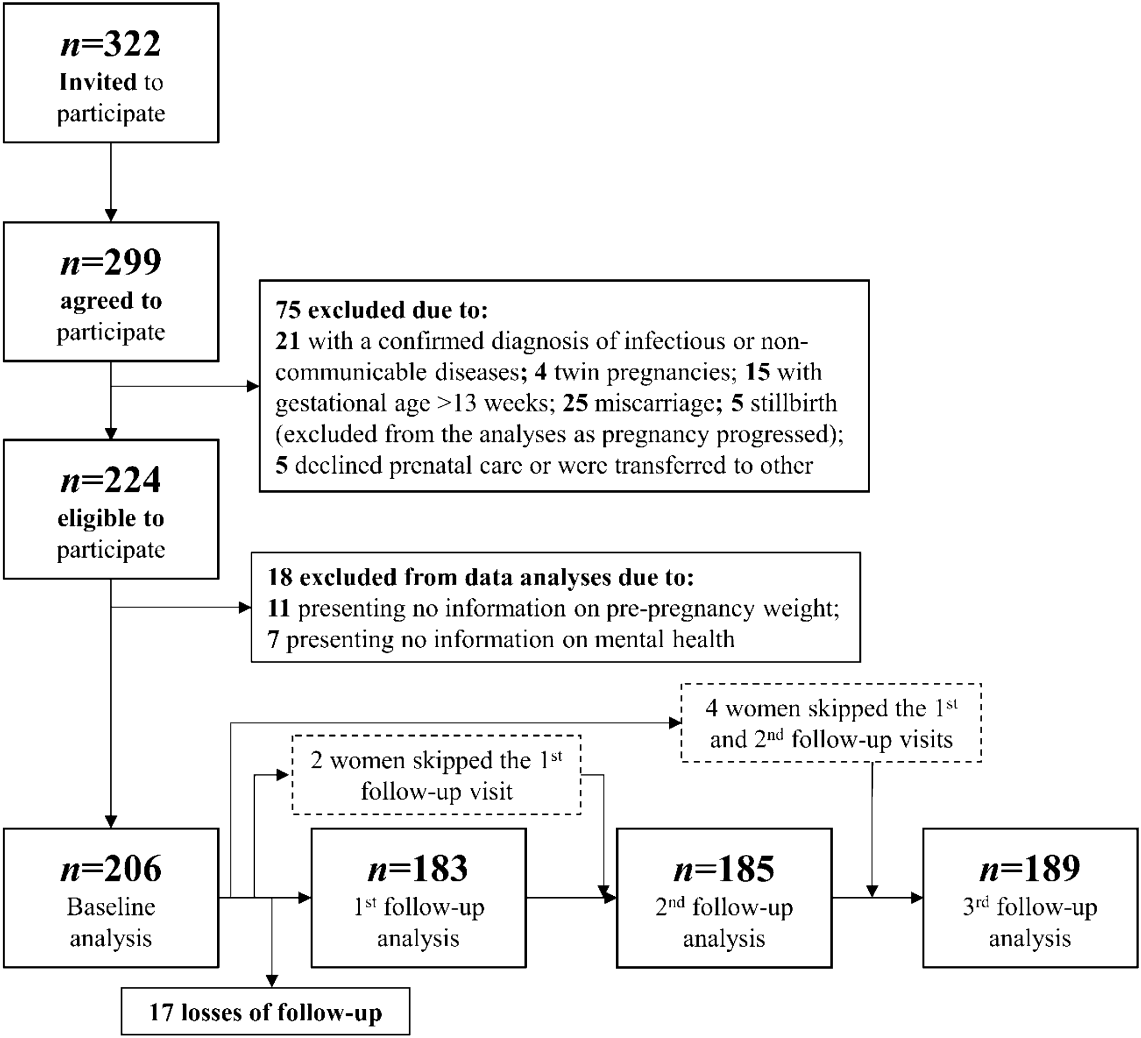
Flowchart to illustrate recruitment process and sample size.

Women were invited to participate if they presented gestational age between 5–13 weeks, age between 20 and 40 years old, and were free of any infectious or chronic non-communicable diseases, except obesity. After enrollment, 299 (93%) pregnant women accepted to participate in the study. Women were excluded if they suffered miscarriages, presented chronic arterial hypertension or multiple gestations, were diagnosed with infectious or non-communicable diseases after the enrollment, abandoned prenatal care, or delivered a stillbirth. For this study, women without information in the mental health questionnaires (n = 7, 3.1%) or without weight/GWG information (n = 11, 4.9%) were also excluded. Thus, we evaluated 206 women in the baseline and 183 in the first, 185 in the second, and 189 in the third follow-up visit (Fig. 2).

### Maternal mental health

Three main instruments were used during pregnancy to evaluate maternal mental health status at different time points: the Edinburgh Postnatal Depression Scale (EPDS), the Spielberger State-Trait Anxiety Inventory (STAI), and the Mini International Neuropsychiatric Interview (M.I.N.I) (Supplemental Table 2).

The EPDS was used in the three pregnancy trimesters (between 5–13, 20–26, and 30–36 gestational weeks) to evaluate depressive symptoms. This scale measures the occurrence of depressive symptoms and contains 10 items, with four response options. Each response can be scored from 0 to 3, and the total score varies from 0 to 30^50^. In this study, a translated and validated version of the EDPS was used^51^, and values ≥ 11 were considered positive for depressive symptoms. Additionally, a variable named ‘persistent depressive symptoms’ was constructed, and women were classified as ‘yes’ if they presented EPDS score ≥ 11 at the three-time points.

The M.I.N.I. (version 5.0.0) was used to assess the presence of major psychiatric disorders, according to the Diagnostic and Statistical Manual of Mental Disorders, 4th edition. The M.I.N.I. is divided into 16 modules (A-P) with a series of questions related to several psychiatric disorders, such as major depressive episodes, generalized anxiety disorder, suicide risk, manic and hypomanic episodes, and obsessive–compulsive disorder. The complete version of the M.I.N.I was used to evaluate the occurrence of major depressive disorder (MDD), generalized anxiety disorder, and the current suicide risk at the first trimester of pregnancy.

Finally, anxiety symptoms were measured using STAI translated and adapted to be used in Brazil^52,53^ at the second (20–26 weeks) and third trimester (30–36 weeks). STAI consists of two distinct scales to measure state and trait anxiety. In the present study, only the state anxiety scale was used. State anxiety refers to a transitory emotional state concerning a specific period. The scale has 20 items with four response options, ranging from one (less anxious) to four (very anxious) points. The total score varies from 20 to 80 points, depending on the intensity of the symptoms. The women with score ≥ 40 points were classified as having anxiety symptoms.

### Gestational weight gain (outcomes)

Maternal anthropometric data were collected according to the standardized protocol established by Lohman, et al.^54^ at the four study visits. Weight (kg) was measured in each trimester on a digital scale (Filizzola PL 150; Filizzola Ltda, São Paulo, Brazil). To determine body mass index (BMI), duplicate measures of height (m) were taken with a portable stadiometer (Seca Ltda, Hamburg, Germany) at the first-trimester interview, and the average of the two measurements was used. Pre-pregnancy BMI (kg/m^2^) was calculated dividing the self-reported pre-pregnancy weight (kg) by the average height (in square meters) and classified according to the World Health Organization^55^ cutoffs as underweight (< 18.5 kg/m^2^), normal (≥ 18.5 and < 25.0 kg/m^2^), overweight (≥ 25.0 and < 30.0 kg/m^2^) and obesity (≥ 30 kg/m^2^).

GWG continuous and categorical were the outcomes of this study. Cumulative GWG was calculated based on the difference between the weight in each visit and self-reported pre-pregnancy weight. Total GWG was classified in below (insufficient), within (adequate), and above (excessive) the IOM^34^ guidelines for each BMI category (Table 4), but considering the woman gestational age at the last weight measurement (37–42 weeks). For example, the highest limit of the adequacy of a woman with a pre-pregnancy BMI between 18.5 and 25 kg/m^2^ that had their last measure of weight at 38 weeks was calculated as 2 kg for the first trimester [13 weeks] + 0.5 kg/week × 25 weeks [38–13 weeks] = 14.5 kg. This way, we considered the gestational age when the last measurement of weight was obtained and to avoid possible misclassifications due to gestational ages different from 40 weeks^56^. In this approach, we considered 0.5 kg of weight gain during the first trimester to calculate the lower limit and 2.0 kg of weight gain in the upper limit.

**Table 4:** Institute of Medicine guidelines for total and rate of weight gain during pregnancy, according to pre-pregnancy BMI.

Prepregnancy BMI	Rates of weight gain 2nd and 3rd trimester^a^	Total weight gain
	Mean (range) in kg/week	Range in kg
Underweight (< 18.5 kg/m^2^)	0.51 (0.44; 0.58)	12.5; 18,0
Normal weight (18.5–24.9 kg/m^2^	0.42 (0.35; 0.50)	11.5; 16.0
Overweight (25.0–29.9 kg/m^2^)	0.28 (0.23; 0.33)	7.0; 11.5
Obese (≥ 30.0 kg/m^2^)	0.22 (0.17; 0.27)	5.0; 9.0

^a^Considering a weight gain range of 0.5; 2 kg in the first trimester. Adapted from IOM^34^.

### Co-variates

Maternal data such as age (years), education (schooling years), monthly per capita family income (< 1/ ≥ 1 minimum wage), marital status (live/does not live with a partner), parity (0 or 1/ ≥ 2 parturitions), desire to be pregnant (yes/no), and practice of pre-pregnancy leisure time physical activity (yes/no) were obtained from structured questionnaires administered by trained interviewers during the first-trimester visit.

Gestational age (GA) was calculated using the date from the first ultrasonography (US) performed prior to 24 weeks of gestation. In cases where the US was unavailable, the date of the last menstrual period was used (n = 14; 6.8%).

### Ethics

The study protocol was approved by the Ethics Committee of Maternity Hospital of Federal University of Rio de Janeiro (protocol: 0023.0.361.000-08) and by the research ethics committee of the Municipal Secretary of Health of Rio de Janeiro city (protocol: 0139.0.314.000–09). All participants signed a term of consent, obtained freely and spontaneously after all necessary clarifications were provided. The study was carried out following the Declaration of Helsinki.

### Statistical analysis

For continuous variables, we described the data using means and standard deviations (SD); for categorical, absolute (n) and relative frequencies (%) were used. We assessed the normality of continuous variables by using histograms and kurtosis and asymmetry measures.

We adjusted linear mixed models with random intercept and slope to evaluate the GWG trajectories during pregnancy according to maternal mental health, including GA as linear, quadratic, and cubic terms. We also tested the interaction between the mental health variables and GA. The coefficients (β) for each of those terms, 95% confidence intervals (CI), and p-values were estimated. For the GWG adequacy, Poisson models with robust variance were adjusted, and incidence rate ratios (IRR) and 95% CI were determined. Effect plots containing the adjusted longitudinal predictions were constructed to graphically represent the longitudinal trajectories of cumulative GWG according to first-trimester major depressive disorder status, persistent depressive symptoms, and anxiety symptoms.

All the multivariate models were adjusted considering the minimal but sufficient set of confounders. The adjustment was selected based on the construction of a directed acyclic graph (DAG), using the web application DAGgity^57^ (Supplemental Figure 1), and considering the literature available on the exposure and outcomes studied. The minimal set of confounders to be included in the models was: desire to be pregnant, schooling years, marital status, pre-pregnancy leisure time physical activity, pre-pregnancy BMI, and parity. The multivariate models aim to evaluate the total effect of maternal mental health disorders on gestational weight gain. When estimating total effects, if one adjusts a model for a mediator, part of the effect of the exposure on the outcome could be not accounted for, and the total effect would be underestimated^58,59^. Therefore, we did not adjust the models for possible mediators, such as dietary intake, nor considered them in the DAG.

The associations between maternal mental health and GWG were considered statistically significant when p-value < 0.05, except for the interaction terms, when a level of significance of 0.10 was used (commonly found in the literature when testing interactions). All the statistical analyses were conducted in Stata (version 15, Stata Corp., College Station, Texas, USA), and R version 3.6 (R Core Team, R Foundation for Statistical Computing Vienna, Austria, 2019) was used to construct the graphs.

## Supplementary Information

Supplementary Information.
