# Supplementary material for: Maternal mental health and gestational weight gain in a Brazilian Cohort
Source: Sci Rep. 2021 May 24;11:10787. doi: 10.1038/s41598-021-90179-6 (PMC8144604; doi:10.1038/s41598-021-90179-6)
Supplement: Supplementary file 1 — Supplementary Information. [file 41598_2021_90179_MOESM1_ESM.pdf]

Title: Maternal mental health and gestational weight gain in a Brazilian Cohort

Authors: Dayana Rodrigues Farias*; Thais Rangel Bousquet Carrilho; Nathalia C Freitas-Costa; Mônica Araújo Batalha; Mylena Gonzalez; Gilberto Kac.

Affiliations:

Nutritional Epidemiology Observatory, Department of Social and Applied Nutrition, Josué de Castro Institute of Nutrition, Federal University of Rio de Janeiro.

Supplemental Table 1. Baseline characteristics of the study population in women considered loss of follow-up and those who completed the study.

	Study participants			
	**Total (n=206) N (%)**	**Completed study (n=189) N (%)**	**Loss of follow-up (n=17) N (%)**	***p***
**Age (years)**				
<30	142 (68.9)	128 (67.7)	14 (82.4)	
≥30	64 (31.1)	61 (32.3)	3 (17.6)	0.279
**Education (schooling years)**				
<8	61 (29.6)	58 (30.7)	3 (17.6)	
≥8	145 (70.4)	131 (69.3)	14 (82.4)	0.200
**Parity (parturitions)**				
0;1	157 (76.2)	144 (76.2)	13 (76.5)	
≥2	49 (23.8)	45 (23.8)	4 (23.5)	0.622
**Pre-pregnancy LTPA**				
No	152 (73.8)	141 (74.6)	11 (64.7)	
Yes	54 (26.2)	48 (25.4)	6 (35.3)	0.374
**Desire to be pregnant**				
No	84 (40.8)	76 (40.2)	8 (47.1)	
Yes	122 (59.2)	113 (59.8)	9 (52.9)	0.582
**Marital status**				
Live with partner	166 (80.6)	153 (81.0)	13 (76.5)	
Does not live with partner	40 (19.4)	36 (19.0)	4 (23.5)	0.748
**Pre-pregnancy BMI (kg/m^2)**				
<25.0	126 (61.2)	117 (61.9)	9 (52.9)	
25.0;29.9	55 (26.7)	50 (26.5)	5 (29.4)	

≥30.0	25 (12.1)	22 (11.6)	3 (17.6)	0.698
**Major depressive disorder**				
No	175 (84.9)	163 (86.2)	12 (70.6)	
yes	31 (15.1)	26 (13.8)	5 (29.4)	0.146
**Generalized anxiety disorder**				
No	185 (89.8)	170 (90.0)	15 (88.2)	
Yes	21 (10.2)	19 (10.0)	2 (11.8)	0.686
**Suicide risk**				
No	165 (80.1)	153 (81.0)	12 (70.6)	
Yes	41 (19.9)	36 (19.0)	5 (29.4)	0.305
**EPDS score**				
<11	132 (65.0)	122 (65.2)	10 (62.5)	
≥11	71 (35.0)	65 (34.8)	6 (37.5)	0.825

Note: *p*-value refers to the chi-squared test

Abbreviations: BMI = body mass index; EPDS = Edinburgh Postnatal Depression Scale; LTPA = leisure time physical activity.

Supplemental table 2. Sample size in each study visit.

	**First trimester (5;13 weeks)**	**Second trimester (20;26 weeks)**	**Third trimester (30;36 weeks)**	**Third trimester (37;42 weeks)**
Gestational weight gain	213	183	187	191
Gestational weight adequacy[a]	*	*	*	190
Depressive symptoms[b]	208	179	185	*
State Anxiety[c]	*	182	185	*
Major depressive disorder[d]	206	*	185	*
Generalized anxiety disorder[d]	206	*	*	*
Suicide risk[d]	206	*	*	*

Note: [a]GWG adequacy according to the Institute of Medicine (2009) guidelines for gestational week; [b]Measured using Edinburgh Postnatal Depression Scale (score ≥11); [c]Measured using the Spielberger State-Trait Anxiety Inventory; [d]Measured using mental health status assessed using Mini-International Neuropsychiatric Interview.

*Not measured.

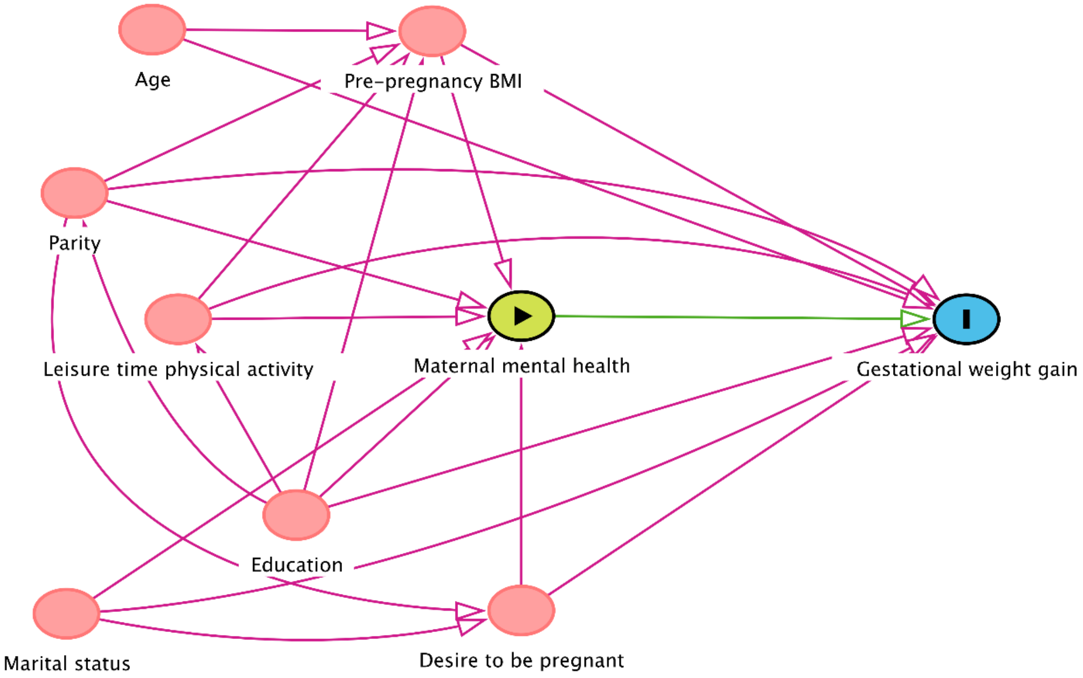

Supplemental figure 1. Directed acyclic graph (DAG) constructed to represent the association between maternal mental health during pregnancy and gestational weight gain.

Note: To estimate the total effect, the minimal set of confounders to be adjusted in the models included: desire to be pregnant, education, leisure time physical activity, marital status, parity, and pre-pregnancy BMI. The DAG was created using the web application 'DAGitty', version 3.0 and is available at [http://dagitty.net/][1].

References

1 Textor, J., van der Zander, B., Gilthorpe, M. S., Liskiewicz, M. & Ellison, G. T. Robust causal inference using directed acyclic graphs: the R package 'dagitty'. *International journal of epidemiology* **45**, 1887-1894, doi:10.1093/ije/dyw341 (2016).
